# Chicago Medical Society

**Published:** 1875-10

**Authors:** D. C. Stillians


### Reports of Societies.

### CHICAGO MEDICAL SOCIETY.

*Regular Meeting*, *Aug*. 16, 1875.

(Reported by D. C. Stillians, M.D.)

Rosa A. Engert, M.D., reported a case of a patient affected with menorrhagia of two or three weeks duration, alternating with intervals, lasting for six or eight weeks, this general condition having existed during the past winter, with severe exacerbations, and no entire relief. The patient was fifty-three years of age, and attributed her disorder to the menopause.

A friable mucous polypoid growth, as large as a plum, was found protruding from the os uteri—the uterus itself being enlarged to the extent of a four months pregnancy. After the removal of the tumor, the wound was cauterized, but, the hæmorrhage continuing, it was found impossible to explore the uterine cavity with the sound. Dilatation of the cervix was effected by the aid of a tent, when the obstruction was found to be occasioned by a soft and spongy mass lying close to the internal os, and easily detected by digital examination. A tampon saturated with a styptic solution was placed in the vagina, when uterine contractions ensued, with increased hæmorrhage as the os dilated, and partial expulsion of the uterine contents. The latter were found to consist of a conglomerate of hyaline cysts, varying in size from a millet seed to a whortleberry, imbedded in a delicate vascular tissue of white stroma. The entire mass was as large as an orange, and was inserted by a pedicle, an inch long and half an inch in width, to the right antero-lateral uterine wall. By the aid of the finger-nails and the curette, the portion of the uterine wall upon which it had been implanted was scraped till the muscular fibres were exposed, when the cavity was syringed with tepid water, and swabbed with dilute solution of acetic acid. Subsequently nitric acid was applied. The morbid growth was evidently a non-malignant uterine papilloma, and its removal was followed by restoration to health.

The reader also reported a case of sarcoma, resulting in well marked encephaloid disease:—

A hard-working woman, 46 years of age, widowed for six years, and having one child born after the death of her husband, had menorrhagia and copious leucorrhœa for one year and a half, the former for six consecutive months. Her strength gradually failed, till she became confined to her bed. She had previously suffered from some ocular disorder, which, under homœopathic treatment, resulted in amaurosis. During this time also, s[illegible] had exhibited a cutaneous exanthem with high fever. A second homœopathic physician had predicated the [illegible]istence of cancer of the womb.

The uppe[illegible] part of the vagina was found filled with a friable colloid mass, readily bleeding when touched. A portion, under examination, seemed destitute of structure, and was thought to be sarcomatous.

The patient was at this time in a cachectic condition, with severe and constant cephalalgia, pain in the back and loins, anorexia, asomnia, chills and fever, followed by diaphoresis, dysuria and constipation. Pulse 120 per minute. There was considerable prostration, with subsultus, and dizziness on assuming the upright position; though she fancied there was slight restoration of vision.

The patient was given beef and eggs, brandy, strychnia, iron, phosphorus and cinchona, until there was improvement in her general condition. Then the accessible portions of the neoplasm were removed, when it was evident that the remnant filled the uterine cavity, with consequent and marked invasion of the tissue of the parietes.

The entire mass was removed by the curette—very rapidly in consequence of the hæmorrhage—when but a narrow border of sound tissue was found remaining. Near the line of junction of the canal of the cervix and the cavity of the uterus, a velvety patch was left unmolested, lest the thinned anterior wall should be perforated in an effort to remove it. The entire uterine cavity was then cauterized with the nitrate of silver.

During the two succeeding days, there was considerable reaction, the pulse rising to 130 per minute, and even more at times, when it was difficult to estimate its rapidity. Diffusible stimulants, with beef tea and opium, were freely administered, and a solution of the permanganate of potash employed locally.

Between five and eight days thereafter, there was a complete amelioration of all symptoms, the pulse falling to 100, and the hæmorrhage entirely ceasing. Menstruation soon recurred at regular intervals, with a slight serous discharge from the womb succeeding each epoch.

Within ten months, a relatively larger growth was found rapidly developing in the same locality. The cells of the latter, on examination, were found to be more numerous and larger than in the first instance. Some of the clusters bore unmistakable resemblance to cancer cylinders.

A *second* operation was refused; the patient complaining merely of slight dysuria, relieved by warm baths and anodynes. Somewhat later, there was exacerbation of pain, delirium, vomiting and death.

The reader was of opinion that an earlier resort to treatment might have secured permanent relief.

Dr. Adolphus concurred in the opinion expressed by the essayist, but stated that hæmorrhage after abortion was not always caused by the presence of a foreign body in the uterus. A lady, under his treatment, aborted at three and a half months, and the secundines were carefully removed—the ordinary discharge continuing about ten days. Her menses appeared at the usual time, and lasted ten days. He found, upon examination, that the uterus was in a normal position, not painful, three and a half inches deep, and that there was ulceration of the cervix. He applied sat. sol. ferri persulphate to the cavity, at intervals of four or five days. The general condition of the patient improved. The menses reappeared and lasted eleven days. He dilated the cervix with sponge tents, and examined the cavity of the uterus thoroughly without finding any foreign body. The lady has had leucorrhœa for several years, and the menorrhagia was doubtless caused by chronic disease. He habitually used, in gynecological practice, the concentrated preparations, introducing them on cotton by means of the applicator, and allowing them to remain till they were expelled.

Dr. Bartlett apprehended some difficulty in reaching the fundus uteri with the index finger, and thought it necessary to introduce half the hand, in order to explore the cavity thoroughly.

Dr. Adolphus. "I can reach the fundus with the index finger by bi-manual palpation in two-thirds of my cases, and in those, where owing to tenderness of the parts, great obesity, or dropsical effusions, I am not able to depress the uterus sufficiently, the use of ether enables me to do so, and explore the cavity with my finger."

Dr. Paoli thought the use of the sat. sol. of perchloride or persulphate of iron, dangerous, producing severe inflammation. Several fatal cases have been reported from their use. Hæmorrhage after abortion, without the presence of a foreign body, is not unusual.

Dr. Engert used these preparations very much diluted, and applied them with a swab of sponge or cotton, to the uterine cavity.

Dr. Quine said that he was constantly using, in the treatment of uterine diseases, the remedies mentioned by Dr. A., which comprised some very active irritants, and escharotics; but notwithstanding this, he held, with Dr. Paoli, that in the cases in which the perchloride and persulphate of iron were employed, and recommended by the essayist, they *were* dangerous—not because they were violently irritating, but because they favored the occurrence of septicæmia by causing the uterus to be filled with blood clots, which were liable to decomposition. Moreover, he had very little confidence in the hemostatic virtues of any mere astringent, locally used, unaided by mechanical agencies. He was generally able to reach the fundus uteri with the finger without introducing the whole hand into the vagina, when the cervix was sufficiently dilated.

Dr. Clark said his views were changing in regard to the use of the preparations just mentioned. He had lately seen three cases of pelvic cellulitis, caused by their use. As in a case lately brought to his notice, where the application was followed by a chill, fever, tenderness over abdomen, long hectic, and a tumor on one side of the pelvic cavity, from which a large quantity of pus was removed.

Dr. Stevenson said that possibly in those cases metrorrhagia where no foreign body was found in the uterus, the result could be explained on "The new basis of Pathology," offered by Prof. King, in the August number of the *Am. Jour. of Obstetrics.*

